# Small RNA-seq analysis of circulating miRNAs to identify phenotypic variability in Friedreich’s ataxia patients

**DOI:** 10.1038/sdata.2018.21

**Published:** 2018-03-06

**Authors:** Marta Seco-Cervera, Dayme González-Rodríguez, José Santiago Ibáñez-Cabellos, Lorena Peiró-Chova, Federico V Pallardó, José Luis García-Giménez

**Affiliations:** 1 Centre for Biomedical Network Research on Rare Diseases (CIBERER- ISCIII), Institute of Health Carlos III, Valencia 46010, Spain; 2 INCLIVA Health Research Institute. Mixed Unit for rare diseases INCLIVA-CIPF. Avenida de Menéndez y Pelayo, 4, Valencia 46010, Spain; 3 Department Physiology. Faculty of Medicine and Dentistry. University of Valencia. Av/ Blasco Ibáñez, 15, Valencia 46010, Spain; 4 EpiDisease S.L. (Spin-Off CIBER-ISCIII) Scientific Park University of Valencia. Av. Agustín Escardino, 9, Paterna 46980, Spain; 5 INCLIVA Health Research Institute. INCLIVA Biobank. Avenida de Menéndez y Pelayo, 4, Valencia 46010, Spain

**Keywords:** Epigenomics, Neuromuscular disease, Next-generation sequencing

## Abstract

Friedreich’s ataxia (FRDA; OMIM 229300), an autosomal recessive neurodegenerative mitochondrial disease, is the most prevalent hereditary ataxia. In addition, FRDA patients have shown additional non-neurological features such as scoliosis, diabetes, and cardiac complications. Hypertrophic cardiomyopathy, which is found in two thirds of patients at the time of diagnosis, is the primary cause of death in these patients. Here, we used small RNA-seq of microRNAs (miRNAs) purified from plasma samples of FRDA patients and controls. Furthermore, we present the rationale, experimental methodology, and analytical procedures for dataset analysis. This dataset will facilitate the identification of miRNA signatures and provide new molecular explanation for pathological mechanisms occurring during the natural history of FRDA. Since miRNA levels change with disease progression and pharmacological interventions, miRNAs will contribute to the design of new therapeutic strategies and will improve clinical decisions.

## Background & Summary

Friedreich’s ataxia (FRDA) is the most common hereditary ataxia, with elevated differences in symptomatology between individuals and within families. Instability of the GAA expansion size is responsible for approximately 50% of the variability found in disease onset^1^. Ataxia stems from spinocerebellar degeneration, peripheral sensory neuropathy, and cerebellar and vestibular pathology, and pyramidal disabilities begin to appear after its onset^2^. Other non-neurological features are related to Friedreich’s ataxia. For example, hypertrophic cardiomyopathy, which is the primary cause of death in these patients, is found very often in FRDA patients. Also, diabetes mellitus^3^ and scoliosis are associated with FRDA^4,5^.

MicroRNAs (miRNAs) are made up of about 22 nucleotides and are the best-characterized small non-coding RNAs (sncRNAs). There is a continuously increasing number of human transcripts corresponding to miRNAs, and their sequences and annotations have been published^6^. miRNA can target mRNAs and control their degradation when there is enough complementarity between the two or when translational repression of protein expression ocurrs^7,8^. Some miRNAs are released from cells in membrane-bound vesicles which protect them from RNase activity^9^, and for this reason miRNAs can be detected in circulating fluids such as plasma, serum, urine and saliva^10–12^. Besides their role in specific tissues, and recently as a stable molecule in circulating fluids^13^, miRNAs have been proposed as biomarkers for some diseases such as cancer^14,15^, diabetes^16^, neurodegenerative diseases^17^, etc.

A small RNA profiling dataset for FRDA patients and healthy controls was generated to identify different miRNA signatures that could explain physiological and molecular pathways underlying this disease and to help determine the phenotypic variability of patients^18^. We found different expression profiles of miRNAs (hsa-miR-128-3p, hsa-miR-625-3p, hsa-miR-130b-5p, hsa-miR-151a-5p, hsa-miR-330-3p, hsa-miR-323a-3p, and hsa-miR-142-3p) between samples from patients and samples from healthy subjects. In addition, we found that hsa-miR323a-3p is a biomarker for phenotypic differentiation in FRDA patients suffering from cardiomyopathy. To the best of our knowledge, this data set represents the largest public small RNA-seq data on a cohort of FRDA patients. The potential for identifying miRNA signatures in FRDA goes beyond the discovery of physiological and molecular pathways underlying this disease. Understanding the phenotypic variability of patients is also necessary for designing the most appropriate therapy for each of them, according to their specific pattern of disease progression.

**Figure 1:**
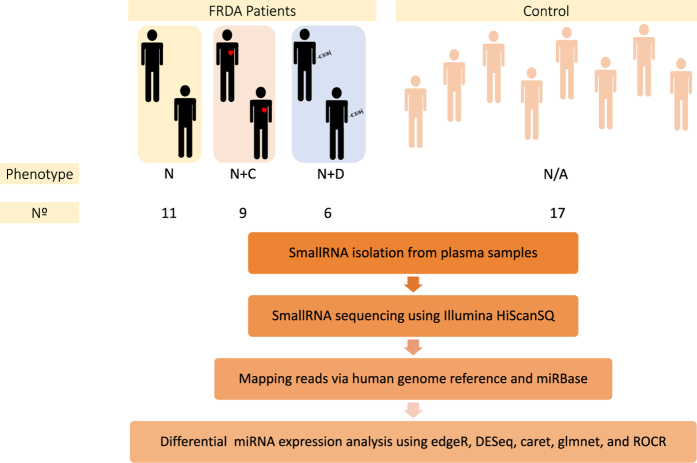
Overview of the study design. Plasma samples from Friedreich’s Ataxia (*n*=25) and healthy subjects (*n*=17) were analyzed. FRDA patients were classified into 3 groups: only neurological disorder (N; *n*=11), neurological disorder plus cardiomyopathy (N+C; *n*=9), and neurological disorder plus diabetes (N+D; *n*=6). One FRDA patient with neurologic symptoms additionally showed both comorbidities, cariomyopahty and diabetes, and thus this patient was classified in both neurological disorder plus cardiomyopathy (N+C), and neurological disorder plus diabetes (N+D) groups. Small RNA from the plasma samples of each FRDA patient and healthy subject was isolated and sequenced to obtain a miRNA expression profile. Next, mapping of the sequencing reads provided whole miRNome status of individual samples. Differential miRNA expression between FRDA patients and healthy subjects and within patients was performed to identify the miRNA signatures of FRDA patients and their concomitant diseases.

In this study, blood samples of FRDA patients (e.g.; FRDA patients with cardiomyopathy “N+C”, FRDA patients with diabetes “N+D”, FRDA patients with only neurological features “N”) and healthy controls were processed and plasma samples were obtained. Plasma samples were used to purify small RNA fractions, then were used to construct the small RNA-seq libraries, and finally were sequenced using the Illumina HiScanSQ platform (Figure 1). The sequence reads were mapped against the human Hg38 build (UCSC Genome Browser). After that, the intersection between the aligned position of the reads and the miRNA coordinates taken from miRBase v21 was performed. In short, we have provided a small RNA-seq data resource on FRDA patients, which is useful to understand phenotypical variability of the disease. Furthermore, the data resource provides an opportunity to identify the biomarkers of diagnosis, prognosis, and treatment monitoring in FRDA.

## Methods

We have already presented some of the methods and tools used in our primary publication^18^. With this paper, we want to expand our previous descriptions and provide a comprehensive resource for reproducing both the experimental and computational analyses. The experimental and analytical procedure we used is described in Figure 1.

### Patient and healthy subject recruitment and clinical features

Patients diagnosed with FRDA without neoplastic diseases or active infection were recruited. Data about age, sex, history of diabetes, cardiopathy, and number of GAA repeats in both alleles were recorded (Table 1; *n*=25). The scale for assessment and rating of ataxia (SARA), a neurological examination-based method to assess the disease, was used to measure the clinical severity of the disease^19^. FRDA patients were enrolled in the study following study approval by the Biomedical Research Ethics Committee (CEIB) of Hospital La Paz (Madrid). Plasma biospecimens from FRDA patients were preserved in a public repository of FRDA in the CIBERER Biobank (www.ciberer-biobank.es; Spanish Biobank Registry number: 000161X02). Healthy volunteers (*n*=25) with no neoplastic diseases, active infection, cardiomyopathy, heart problems, hypertension, or diabetes were enrolled by the Basque Biobank for Research-OEHUN (www.biobancovasco.org) and by the Biobank for Biomedical Research and Public Health of the Valencian Community (IBSP-CV) through the Spanish National Biobank Network (RNBB 2013/12). The subjects of both groups (healthy volunteers and FRDA patients) were matched by sex and age and were processed identically. The selection process and all experimental methods were carried out in accordance with the relevant clinical guidelines, following standard operation procedures, and with the approval of the ethics and scientific committees. All experimental protocols were approved by the Biomedical Research Ethics Committee (CEIB) of Hospital La Paz (Madrid) and the ethics and scientific committees of the IBSP-CV. Informed consent was obtained from all participants.

### Sample collection and small RNA extraction and quantification

Plasma samples were extracted from FRDA patients and healthy participants. For that, blood samples were collected in EDTA tubes and centrifuged at 2500 rpm for 10 min. Once plasma was obtained, each sample was stored at −80 °C until RNA extraction. 500 μL of plasma were used to isolate cell-free total RNA (including miRNAs) using the miRNeasy Serum/Plasma kit (Qiagen, Valencia, CA, USA) following the manufacturer’s protocol. The RNA was eluted with 25 μL of RNase-free water. The concentration of cell-free total RNA (including miRNAs) was quantified using NanoDrop ND 2000 UV-spectrophotometer (Thermo Scientific, Wilmington, DE, USA).

### Small RNA-seq library preparation and sequencing

Small RNA samples were converted to Illumina sequencing libraries using the NEBNext Multiplex Small RNA Library Prep Set for Illumina (Set 1&2) (New England Biolabs, MA, USA), following the manufacturer’s protocol. Briefly, 5′ and 3′ adapters were ligated with small RNA molecules purified from plasma, followed by a cDNA library construction and incorporation of index tags by reverse transcription-PCR (RT-PCR). The products of this RT-PCR were purified using 6% non-denaturing polyacrylamide gel electrophoresis, and then size selection of 145–160 bp fraction was performed. The cDNA library samples were hybridized to a paired end flow cell and individual fragments were clonally amplified by bridge amplification on the Illumina cBot cluster generation. Then, the flow cell was loaded on the HiScanSQ platform and sequenced using Illumina’s sequencing by synthesis chemistry, generating 50 bp single end reads.

### Pre-processing and processing of the reads

**Figure 2:**
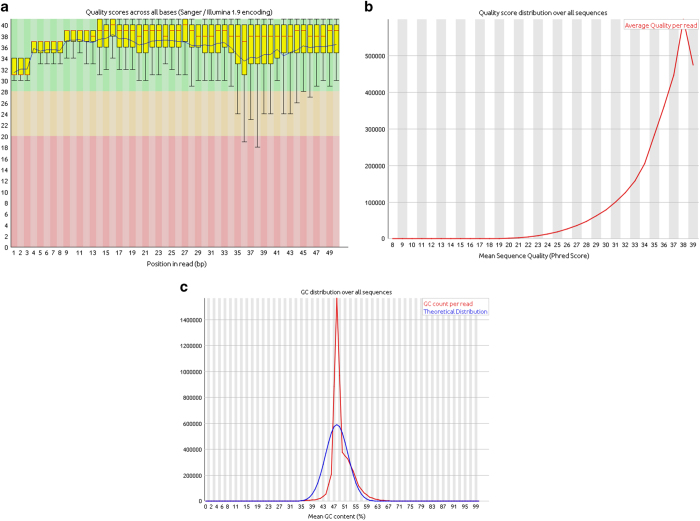
A representative example of quality control metrics of RNA sequenced reads as indicated by FastQC after the preprocessing steps (sample: 11_TAGCTT_L005_R1_001). (**a**) Phred quality score distribution over all reads in each base. (**b**) Quality score distribution over all sequences. (**c**) GC content (%) distribution over all sequences.

The quality of the small RNA libraries was first evaluated using FastQC v0.11.5 software (Figure 2). The most important metrics checked were the overall sequence quality: mean of phred quality per base and per read greater than 30; the GC percentage distribution per read: the data (red curve) is expected to approximately follow the theoretical distribution (blue curve) (Figure 2c). The peaks on the left or on the right side are an indicator of the presence of adapters in the reads. We also checked the presence/absence of overrepresented sequences. Based on the results obtained, the sequence reads were trimmed to remove the following adapter: 5′ AGATCGGAAGAGCACACGTCTGAACTCCAGTCAC- NNNNNN- ATCTCGTATGCCGTCTTCTGCTTG 3′ from each sample. The 6 N’s sequence is unique per sample and allows it to be barcoded. After this step, the bases at the end of the sequences with a quality less than 20 were removed. Finally, all the sequences with length less than 18 nucleotides were discarded. These operations were performed using the tool Cutadapt^20^. The remaining sequences were aligned to the human genome reference (Hg38) from the UCSC Genome Browser. The expression of every miRNA per sample was measured using an annotation file from miRBase (v21). It contained all the mature miRNAs known in humans so far. The alignment and quantification steps were performed using the Subread^21^ and RSubread^22^ packages (http://subread.sourceforge.net/), respectively.

### Differential expression analysis

#### Differential expression analysis between FRDA patients and healthy subjects

Firstly, a differential expression analysis of the miRNAs was performed between patients (*n*=25) and controls (*n*=17). In order to do it, we filtered the miRNAs with less than 1 cpm (counts per million) in 17 samples (the size of the smallest group). Subsequently, we performed a correction of factors using the TMM method^23^ and calculated the effective library sizes. We also estimated the specific dispersions per miRNA with the quantile-adjusted conditional maximum likelihood (qCML) method^24^. The differential expression analysis was executed using the exact test^24,25^. In addition, we carried out a normalization of the original counts estimating the effective library sizes using the geometric mean. The normalized values of the most significant miRNAs (FDR <1e-4) obtained from the differential expression test were used to assess their correlation. Those miRNAs with a level of correlation lower than 0.7 were used to fit a logistic regression model with LASSO penalty^26^. In order to select the most important miRNAs of the model, a leave one out cross validation was performed. Those miRNAs that had non-zero coefficients at the value of λ and that gave minimum mean cross-validated error were selected.

#### Differential expression analysis between FRDA patients grouped by phenotype

We divided the FRDA patients into 3 subgroups according to their phenotype, considering the features described in Table 1. Thus, patients were grouped as 1) patients showing only neurological symptoms (*n*=11); 2) patients showing neurological symptoms “N” and suffering cardiomyopathy “N+C” (*n*=9), and 3) patients showing neurological symptoms and diabetes “N+D” (*n*=6). One FRDA patient with neurologic symptoms additionally showed both comorbidities, cardiomyopathy and diabetes, and thus this patient was classified in both the neurological disorder plus cardiomyopathy (N+C), and neurological disorder plus diabetes (N+D) groups. After this stratification, those miRNAs which did not reach 1 cpm (count per million) in at least 5 samples (size of the smallest group) were filtered out. The data were normalized using the TMM method. Afterwards, a Cox-Reid dispersion^27^ per miRNA was estimated. To find the differentially expressed miRNAs among the three groups compared, the GLM (generalized linear model) ^25^ approach was used. Additionally, we performed new analyses taking into account other variables such as age, sex and disease onset. The last variables were organized as a dichotomy variable according to median values: 37 and 13 years, respectively. Finally, we carried out every comparison between the different groups using the GLM approach. All statistical analyses were performed using R software (version 3.2.2) and the following packages: edgeR (version 3.12.0), DESeq (version 1.22.0), caret (version 6.0–58), glmnet (version 2.0-2), ROCR (version 1.0-7).

### Data Records

RNA-seq data files in FastQ format were deposited at NCBI Sequence Read Archive (Data Citation 1). This accession contains a total of 42 FastQ files resulting from the single end runs for each of the 42 samples. The FastQ format data serves as the raw data from sequencing, which are subjected to further downstream processing. The processed data were deposited at NCBI Gene Expression Omnibus (Data Citation 2).

### Technical Validation

#### Sequencing quality control

We used FastQC v0.11.5 software to perform quality control assessments of the FastQ files before and after the pre-processing steps (filtering, quality trimming and adapter removal). We analysed several measurements, including the overall sequence quality, the GC percentage distribution (i.e. the proportion of guanine and cytosine bp across the reads) and the presence/absence of overrepresented sequences. A representative summary plot after the pre-processing steps is shown (11_TAGCTT_L005_R1_001). Here, the quality scores per base were high, with a median quality score above 30 suggesting high quality sequences across all bases (Figure 2a). The quality score distribution over all sequences was analyzed to see if a subset of sequences had universally poor quality. The average quality for most sequences was high, with scores above 37, which indicated that a significant proportion of the sequences in a run had overall high quality (Figure 2b). The GC distribution per base over all sequences was examined. Despite the GC composition pattern being more similar to the theoretical distribution after the pre-processing steps, it still had a bias (Figure 2c). In addition, overrepresented sequences were examined. Before the adapter removal, some of them were identified as the Illumina indexed adapters used in the sequencing process. After this step, the adapters were no longer identified but we still had overrepresented sequences, possibly because of highly expressed miRNAs (Table 2). All other FastQC files were shown to have similar quality metrics compared to sample (11_TAGCTT_L005_R1_001).

#### Real-time qPCR validation of selected miRNAS from Small RNA-seq

Reverse transcription reactions were performed using the TaqMan miRNA Reverse Transcription kit and miRNA-specific stem-loop primers (Part No. 4366597, Applied Biosystems. Inc, CA; USA) and 100 ng of input cell-free RNA in a 20 μL RT reaction. Real-time PCR reactions were performed in triplicate, in scaled-down 10 μL reaction volumes using 5 μL TaqMan 2x Universal PCR Master Mix (Applied Biosystems. Inc, CA; USA) with No UNG, 0.5 μL TaqMan Small RNA assay (20x) (Applied Biosystems. Inc, CA; USA) [hsa-miR-128-3p (002216), hsa-miR-625-3p (002432), hsa-miR-130b-5p (002114), hsa-miR-151a-5p (002642), hsa-miR-330-3p (000544), hsa-miR-323a-3p (002227), hsa-miR-142-3p (000464), hsa-miR-16-5p (000391)], 3.5 μL of nuclease free water and 1 μL of RT product. Real-time PCR was carried out on an Applied BioSystems 7900HT thermocycler (Applied Biosystems. Inc, CA; USA) programmed as follows: 50 °C for 2 min, 95 °C for 10 min followed by 45 cycles of 95 °C for 15 s and 60 °C for 1 min. We used hsa-miR-16-5p (000391), one of the most stable miRNAs in terms of read counts, and which has been used previously as an endogenous control^27^, to normalize the expression in plasma samples. RNU48 (001006), meanwhile, was used to normalize the expression in cell-line samples. All the fold-change data were obtained using the delta-delta CT method (2^−ΔΔCT^)^28^. The seven differentially expressed miRNAS detected after small RNA-seq were validated using RT-qPCR. All miRNAs were present in plasma at higher levels in patients (*n*=25) compared to healthy controls (*n*=25), in agreement with the results obtained by small RNA-seq. Relative expression levels of the miRNAs in plasma from FRDA patients compared to healthy subjects were shown in Seco-Cervera *et al.*^18^

### Usage Notes

Before processing the raw reads (Data Citation 1) we performed a visual exploration of them by looking for the adapter used in the sequencing process. We saw adapters ligated to the 5′ end for some reads and to the 3′ end in other reads. Despite expecting to always find it at the 3′ end, the opposite situation can sometimes occur. Therefore, we removed the adapter specifying the -b option in Cutadapt. It indicates to the program that the adapter may appear at the beginning (even degraded), within the read, or at the end of the read (even partially).

The alignment can be performed using standard tools, such as Bowtie2^29^, STAR^30^, or Burrows-Wheeler Aligner (BWA)^31^. In our study, we selected the Subread aligner because it is more accurate and faster than previous aligners (nearly four times as fast as the nearest competitor, Bowtie2)^21^. Additionally, the parameters needed when mapping miRNA-seq reads have been well documented. On the other hand, although known miRNA sequences from miRbase can be used as a reference, we suggest using the whole human genome. In this way, the reads aligning to miRNA sequences and to many other features in the genome at the same time can be discarded. The quantification of microRNA expression can be performed using tools like bedtools intersect^32^ or featureCounts^22^. In this step, it is important to allow multiple hits of each read when mapping, since there are multiple copies of some microRNAs in the genome and if it is not allowed, the results might be misleading, or wrong.

Regarding differential expression analysis, we recommend using the popular R packages EdgeR^25^ and DESeq^33^. In the case of using the EdgeR package, it is necessary to filter miRNAs which are not expressed in any condition since they can add some noise to the analysis. Another important aspect to note is the use of an appropriate method according to the different types of comparisons performed. When considering a single study factor, qCML is a good method to estimate the dispersions per miRNA and the exact test to do the differential expression analysis. However, when two or more study factors are included in the analysis, it is highly recommended to estimate dispersions per miRNA with the CR method and to use the likelihood ratio test GLM for differential expression.

## Additional information

**How to cite this article:** Seco-Cervera, M. et al. Small RNA-seq analysis of circulating miRNAs to identify phenotypic variability in Friedreich’s ataxia patients. *Sci. Data* 5:180021 doi: 10.1038/sdata.2018.21 (2018).

**Publisher’s note**: Springer Nature remains neutral with regard to jurisdictional claims in published maps and institutional affiliations.

## Supplementary Material



## Figures and Tables

**Table 1:** Description of FRDA patients and healthy control samples.

**ID**	**Sex**	**Age**	**Neuropathy (N)**	**Diabetes (N+D)**	**Cardiopathy (N+C)**	**GAA repetitions (allele 1/allele 2)**	**Raw Data File**
27	FEMALE	26	1	0	0	885/1150	27_ACAGTG_L007_R1_001
38	FEMALE	32	1	0	1	850/850	38_CCGTCC_L007_R1_001
42	FEMALE	68	1	0	1	75/75	42_GTGGCC_L007_R1_001
41	FEMALE	48	1	0	0	250/250	41_GTGAAA_L007_R1_001
2	FEMALE	39	1	0	0	380/912	2_CGATGT_L005_R1_001
6	FEMALE	56	1	0	1	67/450	6_GCCAAT_L005_R1_001
18	FEMALE	38	1	1	0	500/500	18_CCGTCC_L005_R1_001
5	FEMALE	46	1	0	0	720/950	5_ACAGTG_L005_R1_001
25	FEMALE	46	1	0	1	767/767	25_GTGGCC_L005_R1_001
29	MALE	28	1	0	0	112/112	29_CGTACG_L005_R1_001
1	MALE	34	1	0	1	634/967	1_ATCACG_L005_R1_001
4	MALE	35	1	0	0	700/834	4_TGACCA_L005_R1_001
13	MALE	35	1	0	0	985/985	13_GGCTAC_L005_R1_001
30	MALE	32	1	0	0	480/580	30_GAGTGG_L005_R1_001
14	MALE	41	1	0	0	10/100	14_CTTGTA_L005_R1_001
39	FEMALE	49	1	0	0	314/314	39_GTAGAG_L007_R1_001
17	MALE	47	1	1	0	650/850	17_ATGTCA_L005_R1_001
15	MALE	37	1	0	0	912/1080	15_AGTCAA_L005_R1_001
16	MALE	39	1	1	0	600/734	16_AGTTCC_L005_R1_001
3	MALE	52	1	0	0	185/385	3_TTAGGC_L005_R1_001
26	FEMALE	37	1	0	1	350/980	26_GCCAAT_L007_R1_001
40	FEMALE	37	1	1	0	480/580	40_GTCCGC_L007_R1_001
43	MALE	21	1	1	1	25/25	43_GTTTCG_L007_R1_001
37	MALE	19	1	0	1	1050/1185	37_ATGTCA_L007_R1_001
28	FEMALE	29	1	0	0	634/1000	28_GTTTCG_L005_R1_001
33	FEMALE	24	0	0	0	Not referred to	33_TGACCA_L007_R1_001
34	FEMALE	33	0	0	0	Not referred to	34_AGTCAA_L007_R1_001
49	FEMALE	56	0	0	0	Not referred to	49_CGATGT_L007_R1_001
46	FEMALE	53	0	0	0	Not referred to	46_GGTAGC_L007_R1_001
47	FEMALE	38	0	0	0	Not referred to	47_ATCACG_L007_R1_001
31	FEMALE	44	0	0	0	Not referred to	31_GGTAGC_L005_R1_001
35	MALE	30	0	0	0	Not referred to	35_AGTTCC_L007_R1_001
10	MALE	32	0	0	0	Not referred to	10_GATCAG_L005_R1_001
22	MALE	40	0	0	0	Not referred to	22_GTGAAA_L005_R1_001
7	FEMALE	54	0	0	0	Not referred to	7_CAGATC_L005_R1_001
20	MALE	47	0	0	0	Not referred to	20_GTAGAG_L005_R1_001
21	MALE	39	0	0	0	Not referred to	21_GTCCGC_L005_R1_001
9	MALE	51	0	0	0	Not referred to	9_ACTTGA_L005_R1_001
11	FEMALE	37	0	0	0	Not referred to	11_TAGCTT_L005_R1_001
50	MALE	20	0	0	0	Not referred to	50_TTAGGC_L007_R1_001
44	MALE	16	0	0	0	Not referred to	44_CGTACG_L007_R1_001
45	FEMALE	31	0	0	0	Not referred to	45_GAGTGG_L007_R1_001
0: absence of neuropathy, diabetes or cardiopathy. 1: presence of neuropathy (N), diabetes (N+D) or cardiopathy (N+C).							

**Table 2:** A representative example of quality control metrics of RNA sequenced reads as indicated by FastQC after the preprocessing steps (sample: 11_TAGCTT_L005_R1_001).

**Sequence**	**Count**	**%**	**Possible Source**
AGATCGGAAGAGCACACGTCTGAACTCCAGTCACTAGCTTATCTCGTATG	1264612	41,255	TruSeq Adapter, Index 10 (100% over 49 bp)
GGAAGAGCACACGTCTGAACTCCAGTCACTAGCTTATCTCGTATGCCGTC	88348	2,882	TruSeq Adapter, Index 10 (100% over 50 bp)
GGCTGGTCCGATGGTAGTGGGTTATCAGAACTAGATCGGAAGAGCACACG	53404	1,742	No Hit
TGGAGTGTGACAATGGTGTTTAGATCGGAAGAGCACACGTCTGAACTCCA	24203	0,790	Illumina Multiplexing PCR Primer 2,01 (100% over 29 bp)
AGATCGGAAGAGCACACGTCTGAACTCCAGTCACTTGCTTATCTCGTATG	18397	0,600	TruSeq Adapter, Index 10 (97% over 49 bp)
AGATCGGAAGAGCACACGTCTGAACTCCAGTCACCAGCTTATCTCGTATG	9871	0,322	TruSeq Adapter, Index 10 (97% over 49 bp)
AGATCGGAAGAGCACACGTCTGAACTCCAGTCACTAGCTTATTTCGTATG	9035	0,295	TruSeq Adapter, Index 10 (97% over 49 bp)
AAACCGTTACCATTACTGAGTAGATCGGAAGAGCACACGTCTGAACTCCA	8133	0,265	Illumina Multiplexing PCR Primer 2,01 (100% over 29 bp)
TCCTGTACTGAGCTGCCCCGAAGATCGGAAGAGCACACGTCTGAACTCCA	7712	0,252	Illumina Multiplexing PCR Primer 2,01 (100% over 29 bp)
AGATCGGAAGAGCACACGTCTGAACTCCAGTCACTGGCTTATCTCGTATG	7282	0,238	TruSeq Adapter, Index 10 (97% over 49 bp)
CGCGACCTCAGATCAGACGTGGCGACCCGCTGAATTTAGATCGGAAGAGC	7056	0,230	No Hit
AGATCGGAAGAGCACACGTCTGAACTCCAGTCACTATCTTATCTCGTATG	6722	0,219	TruSeq Adapter, Index 10 (97% over 49 bp)
AGATCGGAAGAGCACACGTCTGAACTCCAGTCACTCGCTTATCTCGTATG	6321	0,206	TruSeq Adapter, Index 10 (97% over 49 bp)
GGCTGGTCCGATGGTAGTGGGTTATCAGAACAGATCGGAAGAGCACACGT	6149	0,201	No Hit
TGGAGTGTGACAATGGTGTTTGAGATCGGAAGAGCACACGTCTGAACTCC	5977	0,195	Illumina Multiplexing PCR Primer 2,01 (100% over 28 bp)
AGATCGGAAGAGCACACGTCTAAACTCCAGTCACTAGCTTATCTCGTATG	5902	0,193	TruSeq Adapter, Index 10 (97% over 49 bp)
GGCTGGTCCGAAGGTAGTGAGTTATCTCAATAGATCGGAAGAGCACACGT	5745	0,187	No Hit
AGATCGGAAGAGCACACGTCTGAACTCCAGTCACGAGCTTATCTCGTATG	5492	0,179	TruSeq Adapter, Index 10 (97% over 49 bp)
AGATCGGAAGAGCACACGTCTGAACTCCAGTCACTAGGTTATCTCGTATG	5167	0,169	TruSeq Adapter, Index 10 (97% over 49 bp)
AGATCGGAAGAGCACACGTCTGAGCTCCAGTCACTAGCTTATCTCGTATG	5163	0,168	TruSeq Adapter, Index 10 (97% over 49 bp)
GGCTGGTCCGATGGTAGTGGGTTATCAGAACCAGATCGGAAGAGCACACG	5022	0,164	No Hit
AGGTCGGAAGAGCACACGTCTGAACTCCAGTCACTAGCTTATCTCGTATG	4697	0,153	TruSeq Adapter, Index 10 (97% over 49 bp)
AGATCGGAAGAGCACACGTCTGAACTCCAGTCACTTGGTTATCTCGTATG	4447	0,145	TruSeq Adapter, Index 3 (97% over 37 bp)
AGATCGGAGGAGCACACGTCTGAACTCCAGTCACTAGCTTATCTCGTATG	4425	0,144	TruSeq Adapter, Index 10 (97% over 49 bp)
GGATCGGAAGAGCACACGTCTGAACTCCAGTCACTAGCTTATCTCGTATG	4249	0,139	TruSeq Adapter, Index 10 (100% over 49 bp)
AGATCGGAAGAGCACACGTCTGAACTCCAGTCACTAGATTATCTCGTATG	4222	0,138	TruSeq Adapter, Index 10 (97% over 49 bp)
AGATCGGAAGAGCACACGTCCGAACTCCAGTCACTAGCTTATCTCGTATG	4136	0,135	TruSeq Adapter, Index 10 (97% over 49 bp)
AGATCGGAAGAGCGCACGTCTGAACTCCAGTCACTAGCTTATCTCGTATG	4022	0,131	TruSeq Adapter, Index 10 (97% over 49 bp)
AGATCGGAAGAGCACACGTCTGAACTCCAGTCACTAGCTCATCTCGTATG	4015	0,131	TruSeq Adapter, Index 10 (97% over 49 bp)
AGATCGGAAGAGCACACGTCTGAACTCCAGCCACTAGCTTATCTCGTATG	3997	0,130	TruSeq Adapter, Index 10 (97% over 49 bp)
GGCTGGTCCGATGGTAGTGGGTTATCAGAACCAGATCGGAAGAGCACACG	5022	0,164	No Hit
AGGTCGGAAGAGCACACGTCTGAACTCCAGTCACTAGCTTATCTCGTATG	4697	0,153	TruSeq Adapter, Index 10 (97% over 49 bp)
AGATCGGAAGAGCACACGTCTGAACTCCGGTCACTAGCTTATCTCGTATG	3929	0,128	TruSeq Adapter, Index 10 (97% over 49 bp)
AGATCGGGAGAGCACACGTCTGAACTCCAGTCACTAGCTTATCTCGTATG	3835	0,125	TruSeq Adapter, Index 10 (97% over 49 bp)
GGCTGGTCCGATGGTAGTGGGTTATCAGAACTTAGATCGGAAGAGCACAC	3818	0,125	No Hit
AGATCGGAAGAGCACACGTCTGAACCCCAGTCACTAGCTTATCTCGTATG	3764	0,123	TruSeq Adapter, Index 10 (97% over 49 bp)
AGATCGGAAGAGCACACGTCTGGACTCCAGTCACTAGCTTATCTCGTATG	3725	0,122	TruSeq Adapter, Index 10 (97% over 49 bp)
AGATCGGAAGAGCACGCGTCTGAACTCCAGTCACTAGCTTATCTCGTATG	3678	0,120	TruSeq Adapter, Index 10 (97% over 49 bp)
AGATCGGAAGGGCACACGTCTGAACTCCAGTCACTAGCTTATCTCGTATG	3599	0,117	TruSeq Adapter, Index 10 (97% over 49 bp)
AGATCGGAAGAGCACACGTCTGAACTCCAGTCACTAGCTTACCTCGTATG	3477	0,113	TruSeq Adapter, Index 10 (97% over 49 bp)
AGATCGGAAGAGCACACGTCTGAACTCCAGTCACTAGTTTATCTCGTATG	3275	0,107	TruSeq Adapter, Index 10 (97% over 49 bp)
AAATCGGAAGAGCACACGTCTGAACTCCAGTCACTAGCTTATCTCGTATG	3132	0,102	TruSeq Adapter, Index 10 (97% over 49 bp)
AGATCGGAAGAGCACACGTCTGAACTCCAGTCACTAGCCTATCTCGTATG	3129	0,102	TruSeq Adapter, Index 10 (97% over 49 bp)
AGATCGGAAGAGCACACGTCTGAACTCCAGTCACTAACTTATCTCGTATG	3127	0,102	TruSeq Adapter, Index 10 (97% over 49 bp)
AGATCGGAAGAGCACACGCCTGAACTCCAGTCACTAGCTTATCTCGTATG	3110	0,101	TruSeq Adapter, Index 10 (97% over 49 bp)
Overrepresented sequences showing count, percentage and possible source for each sequence.			
